# A Review of Autism and the Immune Response

**DOI:** 10.1080/10446670410001722096

**Published:** 2004-06

**Authors:** Paul Ashwood, Judy Van de Water

**Affiliations:** 1 Division of Rheumatology, Allergy and Clinical Immunology Department of Internal Medicine University of California Davis CA USA


*Clinical & Developmental Immunology*, June 2004, Vol. 11 (2), pp. 165–174

Taylor & Francis healthsciences

### A Review of Autism and the Immune Response

PAUL ASHWOOD* and JUDY VAN DE WATER

*Division of Rheumatology, Allergy and Clinical Immunology, Department of Internal Medicine, University of California, One Shield's Avenue, TB162, Davis, CA 95616, USA*

The autistic spectrum disorders (ASD) are complex developmental disorders that manifest during childhood. By definition, children with ASD are characterized by qualitative impairments in social interaction, deficits in verbal and non-verbal communication, and with restricted repetitive and stereotyped patterns of behavior and interests (American Psychiatric Association, 1994). ASD describes a range of conditions including autism and Asperger's syndrome that fall within the umbrella term of pervasive developmental disorders (PDD). The prevalence of ASD has increased substantially over the last decade rising from 5 to 60/10,000 (Fombonne, 1999; Bertrand *et al.*, 2001; Chakrabarti and Fombonne, 2001; Fombonne *et al.*, 2001; Wing and Potter, 2002). The male to female ratio in autism are also skewed with a three- to four-fold increased incidence in boys. In the majority of cases the cause of ASD are unknown, although strong genetic links have been shown for cases with tuberous sclerosis, neurofibromatosis, chromosomal abnormalities and fragile X. In addition, environmental factors have been implicated for prenatal rubella infection, anticonvulsants and antiemetics taken during pregnancy, perinatal hypoxia, post-natal infections such as encephalitis and in metabolic disorders such as phenylketonuria (Fombonne *et al.*, 2001; Baird *et al.*, 2003). Evidence for the contributing role of genetic factors in ASD have also come from monozygotic twin and family studies, with concordance rates for broad spectrum ASD of 90% and a familial risk of 5–10 times higher than the general population, respectively (Bailey *et al.*, 1995; Szatmari, 1999; Rutter, 2000). However, within the general population there appears to be no bias or selective pressure against ASD traits, where arguably from the evolutionary viewpoint due to impairments in social interactions and in some cases lifelong dependency, one would anticipate selective pressure would exist. As such, it has been suggested through genomic screens, that in autism there are multiple weak gene interactions. Indeed, many candidate genes that determine disease susceptibility, or severity, or disease course, have been implicated and studied in autism. There exists a great variability in gene expression (Szatmari, 1999; Risch *et al.*, 1999; Rutter, 2000), with the most likely genetic models predicting multiple gene interactions of up to 15 or more with low individual contributions. However, unequivocal evidence for a genetic association with ASD has so far not been established. Moreover, with the increased prevalence of autism the influence of a definitive set of genetic determinants has become more difficult to determine. The lack of association between ASD and social class, immigrant status and geographical location may suggest that a biological i.e. not solely genetic cause, plays a leading role in the etiology of autism. Furthermore, features such as epilepsy, clear clinical regression of development, mental retardation, persistent neurological signs such as primitive reflexes (Volkmar *et al.*, 2003) and gastrointestinal symptoms occur in some but not in all cases. Thus there may exist subsets of ASD patients, with separate multiple etiologies that affect biological and psychological functioning, that manifest with differing clinical phenotypes.

There is a growing awareness of an immunological involvement in children with ASD. Systemic immunologic aberrations in ASD have been linked with both autoimmunity, describing antibodies reactive for CNS proteins with the potential for neuronal tissue destruction, and secondly, with dysfunctional immunity such as abnormalities or deficits of function in immune cell subsets, leading to an inappropriate or ineffective immune response to pathogen challenge. Indeed, in general the links between the immune and neurological systems are becoming increasingly well known. Products of the immune system may play an important role in early neurodevelopment and can influence patterns of behavior. The immune and nervous system are both complex, highly evolved systems preserved within structural networks that transfer signals through the release of chemical mediators such as neuropeptides and cytokines. In addition, the neuronal synapse and the contact interface between T cells and antigen presenting cells share a structurally similar

*Corresponding author. Tel.: +1-530-752-3285. Fax: +1-530-752-6047. E-mail: pashwood@ucdavis.edu

ISSN 1740-2522 print/ISSN 1740-2530 online © 2004 Taylor & Francis Ltd
DOI: 10.1080/10446670410001722096

architecture. There is continual cross communication between the immune and nervous systems, with many peptides playing a role in both. In ASD, through the analysis of neuropeptide and neurotransmitter levels, many neuroactive compounds that also share immunomodulatory properties, have been implicated in the disease process.

### NEUROTRANSMITTERS AND NEUROIMMUNOLOGY

Neuropathologic findings in autism include reduced numbers of neurons and reduced dendritic arborisation in the amygdala, hippocampus, mammillary bodies and medial septial nucleus (Kemper and Bauman, 2002). In addition, observations of increased brain size compared with controls and decreased numbers of Purkinje and granule cells have also been noted in autism (Volkmar *et al.*, 2003). These findings clearly indicate that there are neurological involvements in ASD that affect the development and differentiation of neurons in the brain. It has been proposed that increased or abnormal levels of neuroactive compounds early during neurodevelopment may lead to autism. Indeed, abnormalities in various neurotransmitter systems have been implicated in the development of autism, including serotonin, dopamine, noradrenaline (norepinephrine), gamma-aminobutryic acid (GABA), glutamate and neuropeptides. Such peptides as oxytocin and vasopressin which are involved in immune tolerance, serotonin (5-HT) which exerts concentration dependent suppressive or proliferative responses on T cells, and the immunomodulating vasoactive intestinal peptide (VIP), have all been implicated in ASD (Nelson *et al.*, 2001; Sharp, 2003).

Serotonin is a biogenic amine that has wideranging effects on numerous physiological processes such as circadian rhythyms, appetite, mood, sleep, anxiety, motor activity and cognition. Serotonin is found in serotonergic neurons of the nervous system, enterochromaffin and neuroendocrine cells of the endocrine system as well as in platelets and on lymphocytes of the immune system. Consistent findings of increased peripheral blood platelet serotonin levels have been demonstrated in approximately one third of children with autism (Anderson *et al.*, 1990; Singh *et al.*, 1997), while selective 5-HT reuptake inhibitors have been shown to be beneficial with regard to treating obsessional and repetitive behavioral phenotypes in some patients (McDougle *et al.*, 1996). The mechanism by which platelet 5-HT is elevated remains unknown, but it is likely that the effect is due to alterations in the platelets themselves resulting in increased platelet uptake or reduced platelet 5-HT2 receptor binding (Cook and Leventhal, 1996). Interestingly, auto-antibodies directed toward serotonin receptors have been found in autistic patients compared with controls (Todd and Ciaranello, 1985; Singh *et al.*, 1997), suggesting an abnormal immune response may be involved in serotonin regulation. In addition, enzymes that control the conversion of tryptophan into serotonin or the formation of kynurenine derivatives are under the control of cytokines such as IFN$\gamma$ and IL-1 (Dunn *et al.*, 1999; Wirleitner *et al.*, 2003). Typtophan depletion has previously been shown to result in an increase of autistic behaviors (McDougle *et al.*, 1996). Thus, accelerated tryptophan degradation as a result of immune activation could lead to an increase in autistic features. In autistic boys, decreased measurements of serotonin synthesis in the frontal cortex and thalamus, but elevated synthesis in the contralateral dentate nucleus were observed using PET scans (Chugani *et al.*, 1999). However, factors such as the age or gender of the subject and associated mental retardation may alter serotonin levels. Nonetheless it has been proposed that there exists a normal developmental process characterized by high serotonin levels in typically developing children up to 5 years of age, which is disrupted in autistic children (Chugani *et al.*, 1999). Encouraging preliminary data have suggested a link between the 5-HT transporter gene and autism, but so far the nature of the specific link remains elusive (Anderson *et al.*, 2002; Conroy *et al.*, 2004; Nabi *et al.*, 2004). Similarly, changes in blood levels of norephinephrine, aspartic acid, glutamine, glutamic acid and GABA have been shown in individuals with autism compared with controls (Cook *et al.*, 1990; Rolf *et al.*, 1993), however, further large scale studies with patients matched for age and behavioral characteristics need to be performed.

Opioid peptides and opioid receptors are important modulators of neural development, influencing migration, proliferation and differentiation within the CNS (Zagon, 1987). It has been hypothesized that an excess of opioid peptides will have detrimental effects on brain development and behavior, and that autism may be a result of abnormal levels or activity of opioid peptides. Not only are immune cells capable of producing opioid peptides, but they also have opioid receptors on their cell surface. The precise mechanisms that underlie the immunosuppressive effects of opioids remain unknown, however, it has been proposed that they can operate as cytokines (Peterson *et al.*, 1998), acting through receptors on peripheral blood and/or glial cells. For example, the anti-inflammatory kappa-opioid drugs are able to reduce adhesion molecule expression, inhibit cellular trafficking, reduce TNF-*a* release and expression, and alter mRNA expression of neuropeptides, presumably through opioid–receptor interactions involved in different phases of an immune response (Walker, 2003). In contrast, beta-endorphin increases the expression of complement and Fc$\gamma$ receptors on the surface of neutrophils and induces concentration dependent stimulation of reactive oxygen intermediates (Menzebach *et al.*, 2003). The actions of beta-endorphin on neutrophils are mediated through the N-terminal fragment, and not the C-terminal fragment. Studies on beta-endorphin in autism have found increased C-terminal and reduced N-terminal beta-endorphin fragments (Leboyer *et al.*, 1994). However, a clear pattern of beta endorphin levels has yet to be established as further studies have found increased, decreased and normal levels in plasma and CSF (Croonenburghs *et al.*, 2002). Another important source of opioids with neurmodulating activity is from the diet. Beta-casemorphine-7, an opioid exclusively of dietary origin, has been shown to be present in patients with pyschoses including autism (Wakefield *et al.*, 2002). Indeed, the beneficial effects on autistic behavior following dietary exclusion therapy are thought, in part, to be a result of reduced opioid intake (Knivsberg *et al.*, 1995; Whitely *et al.*, 1999; Knivsberg *et al.*, 2002). Furthermore, therapeutic trials using the oral opioid antagonist, naltrexone, in autism have shown improvements in behavioral characteristics such as repetitive stereotypies, social contact, and self-injury (Herman *et al.*, 1987; Panksepp *et al.*, 1991; Zingarelli *et al.*, 1992). How the immune system is affected by these therapeutic interventions is largely still unknown.

Neuropeptides are small, biologically active peptides derived from the central and peripheral nervous system. Through animal models, it has been suggested that the neuropeptides oxytocin and vasopressin play a critical role in the regulation of social recognition, affiliation and attachment (Young *et al.*, 2002). Prairie voles are social animals who form long lasting pair bonds. Central infusion of oxytocin in females or vasopressin in males helps to establish partner bonding, a phenomenon that can be blocked using specific antagonists (Williams *et al.*, 1994; Young *et al.*, 2001). In prairie voles, oxytocin and vasopressin receptors are located in the ventral forebrain whereas, in contrast, asocial or solitary montane voles which do not form bond pairs have different patterns of expression for oxytocin receptors (Young *et al.*, 2002). Therefore, the pattern of receptor distribution together with the concentration of released neuropeptides are important in the establishment of socially rewarding interactions. Furthermore, oxytocin knockout mice have normal cognitive abilities but have diminished social recognition (Ferguson *et al.*, 2000). In autism, plasma levels of oxytocin are reported to be significantly lower than controls (Modahl *et al.*, 1998). In addition, further analysis of neuropeptides in neonatal blood by recycling immunoaffinity chromatography found that concentrations of VIP, calcitonin-gene related peptide, brain-derived neuurotrophic factor and neurotrophin 4/5 were increased in ASD compared with typically developing control children but could not be distinguished from those with mental retardation (Nelson *et al.*, 2001). So far, signature patterns of neurpeptides and neurotransmitters in autism have yet to be established.

It is increasingly clear that besides their function as neurotransmitters, neuropeptides have an influence on almost all body functions including the immune system. For example, oxytocin is present in the thymus at high levels and is expressed on thymic epithelial cells, where it is thought to act both as a self-antigen and as a promoter of T cell focal adhesion (Gimpi and Fahrenholz, 2001) and as such plays a role in immune tolerance. In addition, Dunzendorfer *et al.* have suggested a role for neuropeptides as immunomodulators and have presented a novel concept of neuropeptide-mediated regulation of dendritic cell (DC) migration. They investigated locomotion of mononuclear cell-derived DCs at different maturation stages toward gradients of sensory neuropeptides *in vitro*. Calcitonin gene-related peptide, VIP, secretin, and secretoneurin induced immature DC chemotaxis comparable to the potency of the chemokine RANTES, whereas substance P and macrophage-inflammatory protein-3 beta stimulated immature cell migration only slightly. Moreover, there was a distinct neuropeptide-induced signal transduction seen in DC exposed to neuropeptides (Dunzendorfer *et al.*, 2000). In addition, it has been shown that VIP synergizes with TNF-$\alpha$ in the induction of DC maturation (Delneste *et al.*, 1999). The notion that a defect in DC chemotaxis could have ramifications on the normal process of immature DC migration to sensory nerve fibers where they participate in inflammatory events is intriguing in the context of autism. In the central nervous system (CNS) DCs have been found in normal meninges, the choroid plexus, and cerebrospinal fluid. During inflammation in the CNS, there is a recruitment of DCs. It has been speculated that they may equally play a role in the defense against infections and contribute to the break-down of tolerance to CNS autoantigens (Pashenkov *et al.*, 2003). Microglia cells, another CNS cell lineage, are antigen presenting cells (APCs) that share similarities with both DCs and macrophages. Microglia cells, as part of the CNS innate immune system, participate in many reactive processes in the CNS and have been implicated in the exacerbation of neurological conditions such as multiple sclerosis, Alzheimer's disease, AIDS, and viral encephalitis (Nelson *et al.*, 2002). In the autoimmune encephalomyelitis (EAE) model and in patients with multiple sclerosis, self-myelin antigens were observed in cells expressing DC/macrophage markers, MHC class II, and co-stimulatory molecules, a finding not present in controls (de Vos *et al.*, 2002). Taken together, these findings suggest a central role for DC and neuropeptide mediated chemotaxis in the control of CNS inflammation and modulation of T cell reactivity against CNS antigens.

Cytokines and other products of immune activation have widespread effects on neuronal pathways, and in part may be responsible for many common features of ASD such as mood and sleep disturbances. The brain is protected and isolated by the blood brain barrier from potentially deleterious agents present in the blood including inflammatory immune cells and proteins. However, recent data have suggested that the brain is an immunologically active organ and that immune reactions do occur within the CNS. The entry of cytokines produced in the periphery into the brain is predominantly restricted by the blood brain barrier. However, they may gain access through active transport mechanisms or at circumventricular regions where the barrier is less controlling (Wilson *et al.*, 2002). In addition, cytokines may affect brain barrier function directly by binding to receptors on brain endothelial cells leading to the impairment of function (Wilson *et al.*, 2002). Evidence has emerged that leuckocytes migrate into the brain from the blood via the cerebral spinal fluid to the choroids plexus, or from the blood to either the subarachnoid space or parenchymal perivascular space (Ransohoff *et al.*, 2003). These leuckocytes may then act as a potential source of cytokines. Alternatively, afferent neurons may be directly responsive to peripheral cytokine stimulation (Dantzer *et al.*, 1998). Indeed, cytokines can affect enteric glial cells (Von Boyen *et al.*, 2004). The effects of cytokines on the mood, sleep, nutritional intake, exploratory behavior, and social interactions do not necessarily have to result in neuronal damage or death. Systemic cytokines administered at therapeutic doses, such as IFN-α, IL-2 and TNF-α, have side effects including mood depression, sleep disorder, impaired cognitive function, decreased exploratory behavior, and changes in motivation (Licinio *et al.*, 1998; Larson, 2002). Systemic cytokine administration can also cause increases in noradrenergic, dopaminergic and serotonergic metabolism in the hypothalamus, hippocampus and nucleus accumbens (Mohankumar *et al.*, 1991; Shintani *et al.*, 1993; Merali *et al.*, 1997). Cytokines can activate and exert trophic effects on glial cells, and in turn, glial cells can produce cytokines and chemokines upon activation. As the CNS is largely populated by astroglia and microglial cells, these cytokine–cell interactions have important implications for neuronal cell functioning and development.

### SYSTEMIC IMMUNE ABNORMALITIES IN ASD PATIENTS

Various immune abnormalities have been described by an increasing number of laboratories worldwide with findings consistently showing both the involvement of autoimmune processes and the dysregulation of the immune response in children with autism. Many autistic children suffer prolonged and recurrent infections, as well as reduced responsiveness to common recall antigens on cutaneous delayed hypersensitivity tests compared with controls (Murch *et al.*, 1999). Immune aberrations consistent with a dysregulated immune response reported in ASD children include decreased peripheral lymphocyte numbers (Ashwood *et al.*, 2003), decreased response to T cell mitogens (Stubbs and Crawford, 1977; Warren *et al.*, 1986), incomplete or partial T cell activation evinced by increased numbers of DR+ T cells without the expression of the IL-2 receptor (Plioplys *et al.*, 1994; Denney *et al.*, 1996), dysregulated apoptosis mechanisms (Engstrom *et al.*, 2003), and the imbalance of serum immunoglobulin levels including increased IgE and decreased serum IgA (Ashwood *et al.*, 2003). In initial cytokine analysis studies of 10 autistic children Stubbs (Stubbs, 1995) showed increased levels of plasma IFN-α compared with 4 adult controls. In a further study, plasma levels of IL-12 and IFNγ were increased in autistic children but there were no differences between IL-6, TNFα and IFNα when compared with controls (Singh, 1996). These studies proposed that there are aberrant T cell cytokines profiles in children with ASD compared with controls. However, there is an apparent divergence of opinion on the predominant polarity of the dysregulated immune response. In cell culture studies, Gupta showed intracellular cytokine levels of stimulated IL-2 and IFNγ expressing CD4+ and CD8+ cells to be lower in 20 autistic patients compared with 20 aged matched controls. In contrast, the number of CD4+ and CD8+ cells expressing IL-4 were increased (Gupta *et al.*, 1998). Croonenburghs *et al.* (2002) reported increased IFNγ, IL-6 and IL-1ra in unstimulated culture supernatants from ASD patients, although no information on experimental design or details of autistic patients or controls were given. More recently, plasma IFNγ levels in 29 autistic children correlated positively with the generation of the potent functional and developmental intercellular CNS messenger nitric oxide (NO), a finding indicative of NO-related oxidative stress (Sweeten *et al.*, 2004). A similar mechanism of IFNγ induced plasma neopterin, may be responsible for the elevated levels observed in autistic patients and may reflect cell-mediated immune activation (Sweeten *et al.*, 2003). Evidence for an increased inappropriate innate immune response was shown by the over-production of pro-inflammatory cytokines, in particular TNFα, in peripheral blood mononuclear cells stimulated with bacterial LPS in ASD children compared with controls (Jyonouchi *et al.*, 2001). Interestingly, siblings also showed raised TNFα levels compared with controls suggesting a genetic defect in control of innate immune responses in children with ASD and their family members. Furthermore, decreased NK cell activity has been shown in ASD children and is further indicative of a functional deficit in the innate immune response (Warren *et al.*, 1987). However, very few studies on the innate system in patients with autism have been performed thus far. Other findings in autistic subjects, with an immunological significance, include increased frequency of the null alleles of complement and HLA-DRB1*04 allele and low levels of C4b complement protein (Warren *et al.*, 1991, 1994, 1996). However, it is hard to ascertain a clear signature immune cellular or cytokine response in the pathology of early childhood development seen in autism. So far there are a lack of systematic studies with sufficiently large enough sample sizes. In addition, many studies are not adequately matched for the age and gender of subjects and controls, where autistic children are often compared with much older controls. In part, these difficulties occur as there is often limited access to autistic populations and the behavioral aspects of autism make sampling difficult. Furthermore, the young age of the subjects makes rigorous and wide-ranging immunological studies prohibitive. A further consideration is that it is now becoming increasingly apparent that autistic patients may be categorized into various phenotypes based on differing patterns of genetic, immunologic and behavioral aspects. In the future, well defined studies with fully characterized patient and matched control groups will need to be performed in order to ascertain how the immune response is involved in autistic pathology.

### AUTOIMMUNE PROCESS IN ASD

The presence of antibodies directed against components of the CNS in the sera of autistic children, is indicative that a possible autoimmune process may be involved in the pathology of ASD. Autoimmune diseases arise when the immune system is inappropriately directed to recognize and exert an exaggerated respond to "self" components. These include, but are not restricted to diseases such as; myasthenia gravis, multiple sclerosis, systemic lupus erythematosus, primary biliary cirrhosis, and Graves thryrotoxicosis. The exact mechanism of autoimmunity in these diseases is not identical, but they all possess autoreactive antibodies and T cells. Various anti-brain antibodies have been found in autistic patients including autoantibodies to serotonin receptor (Todd and Ciaranello, 1985), myelin basic protein (MBP) (Singh *et al.*, 1993), neuron axon filament protein (NAFP) (Singh *et al.*, 1997), cerebellar neurofilaments (Plioplys *et al.*, 1994), nerve growth factor (Kozlovskaia *et al.*, 2000), alpha-2-adrenergic binding sites (Cook *et al.*, 1993), anti-brain endothelial cell proteins (Todd *et al.*, 1988; Connolly *et al.*, 1999) and antibodies against the caudate nucleus (Singh and Rivas, 2004). In a recent paper, serum from a mother with an autistic child found to bind to Purkinje cells and other neurons, when injected into gestating mice, induced behavioral changes including altered exploration, motor coordination and changes in cerebrallar magnetic resonance spectroscopy in the offspring. In contrast, mice injected with sera from mothers with typically developing children showed no behavioral changes (Dalton *et al.*, 2003). This study supports the notion that maternal antibodies may influence the neurodevelopmental process in autism. In addition, the frequency of autoimmune disorders in family members of 61 ASD children and 46 typically developing normal controls were compared (Comi *et al.*, 1999). The frequency of autoimmune disorders was increased in the ASD group with over 40% of families having two or more close family members with autoimmunity. Findings that have since been replicated by Sweeten *et al.* (2003) in families of pervasive developmental disorder probands. However, these studies were based on self-reporting rather than medical records and thus may have a significant margin of reporting error.

The pathophysiological significance of auto antibodies reported in children with autism is uncertain. For example increased autoantibodies would suggest that there is increased neuronal damage, as is the case in multiple sclerosis where, following demyelination, MBP is unmasked and there is a subsequent generation of antibodies. To date however, evidence of demyelination in autism has remained elusive (Rumsey and Ernst, 2000). Glial fibrillary acidic protein (GFAP) measured in the CSF of 47 autistic children was significantly elevated compared to age matched 10 control children, suggesting that gliosis and unspecific brain damage may occur in autism (Ahlsen *et al.*, 1993). However, as GFAP correlates strongly with age, due most likely to age dependent expansion of fibrillary astrocytes, caution must be shown in interpreting these data (Rosengren *et al.*, 1992). Another possible cause for the development of autoimmunity is a dysfunction in immune regulation, that allows immune responses to continue unchecked. One regulatory T cell population is identified by the combination of the CD4 (T helper cells) and CD25 (IL-2 receptor) cell surface markers, which are a cell population that as discussed above, is at a low frequency in autism. A dysregulation of IL-10 and TGF$\beta$1 release parallel to times when pro-inflammatory signals such as TNF$\alpha$ are high, will lead to an imbalance of inflammatory signals. Furthermore, defects in apoptosis mechanisms will promote the survival of immune cells including autoreactive lymphocytes. Dysregulated FAS mediated cell death and increased soluble FAS receptor have been described in autism (Engstrom *et al.*, 2003). Observations of elevated anti-CNS antibodies in autism, are at best unconfirmed, and in some cases, for example serotonin receptors and MBP, markedly conflicting. However, taken together the findings of autoimmunity in families and the plethora of anti-brain antibodies suggest that in some patients, autoantibodies that target the CNS may be a pathological or exacerbating factor in neuronal development in children with ASD. It is also important to note that for each antibody tested the number of autistic children showing positivity was far from 100%. It is also unclear whether individual autistic children are positive for more than one antibody. Again, it can be inferred that increased autoimmunity may be confined to a subset of autistic patients. Indeed, large cohort studies with thoroughly defined and specifically phenotyped autistic patient groups and well matched age and sex controls, need to be performed to confirm the potential role of autoantibodies in the pathology of either all or subsets of autistic patients. Moreover, the development of an animal model will be crucial to determine the role of autoantibodies in the pathology of autism.

### RESPONSE TO VIRUSES

Many infectious agents including; rubella, measles, human herpesvirus 6 (HHV-6), influenza and cytomegalovirus have been associated with the aetiology of autism (Chess *et al.*, 1978; Binstock, 2001; Yamashita *et al.*, 2003). These associations, although not conclusive for all children with ASD, may highlight an underlying inability of ASD children to fully eradicate viral insults. As such, viruses and viral products may persist in children with ASD. Indeed, defects in T cell numbers and function, a phenomenon observed in ASD, may compromise the body's defense system to infectious agents and engender the body susceptible to recurrent infections. Persistent viral infection has been described in mucosal tissue of ASD children compared with age-matched typically developing controls, with viral RNA present in DC populations (Uhlmann *et al.*, 2002). This persistence may be indicative of a primary failure to successfully eradicate antigen and the inability to deal with viral insults. Furthermore, increases in lymphoid tissue seen at mucosal sites in ASD children may be reflective of a local inflammatory response to the presence of a persisting Ag (Wakefield *et al.*, 2000; Ashwood *et al.*, 2003). Paramyxoviruses, such as measles have profound effects on DC function including the inhibition of LPS stimulated IL-12 release. In addition, the ability to stimulate T cell proliferation by mitogen, was greatly reduced in the presence of measles infected DCs (Klagge and Scheider-Schaulies, 1999). The migration of infected DCs to T cell areas of lymphoid organs could therefore exert immunosuppressive effects on the surrounding T cells.

In a mouse model, viral infections in pregnant mothers leave long-lasting effects on the offspring, including an increased risk of behavioral abnormalities with particular relevance to autism and schizophrenia (Patterson, 2002). In pregnant mice (BALB/c and C57BL/6 strains), when human influenza virus was administered, offspring displayed aberrant behavioral responses as adults, with deficits in prepulse inhibition (PPI) in the acoustic startle response, findings also observed in autistic subjects (Fatemi *et al.*, 2002). It is hypothesized that maternal immune responses generated to eradicate the viral insult affect fetal brain development and the upregulation of specific cytokines are implicated at this time. In another animal model, infection with Borna disease virus (BDV) in Lewis rats leads to behavioral and CNS abnormalities reticent of neurodevelopmental disorders such as schizophrenia and autism in humans (Hornig *et al.*, 1999). Borna disease virus infection primarily targets the hippocampal neurons, affecting ERK 1/2 phosphorylation, the expression of synaptic vesicle proteins, and hampers synaptogenesis and synaptic organization induced by the neurotrophin brain-derived neurotrophic factor (BDNF). However, BDNF receptor expression, neuronal survival and morphology are not affected (Hans *et al.*, 2004). These findings show that BDV acts in a non-cytopathic way to alter neuronal growth factor mediated events and thereby alter synaptic connections between neurons, possibly by interfering with cell signaling events downstream of receptor binding, where such alterations may affect all functions of the brain. These models of infection will provide valuable insight into the mechanisms of human neurodevelopmental disorders and the interplay of the developing neuronal and immune system in the pathology of environmentally induced behavioral abnormalities.

Bacterial and viral infections have been suspected in the triggering of or excacerbation of autoimmune diseases (Panoutsakopoulou and Cantor, 2001). In autism, patients with anti-measles IgG antibodies were also positive for anti-MBP (90%) and anti-NAFP (73%) antibodies, a similar relationship were seen in patients with positive titers for anti-HHV-6 antibodies and corresponding anti MBP and anti-NAFP antibodies (Singh *et al.*, 1998). Interestingly, in these studies anti-measles and anti-HHV-6 antibodies were not different between autistic patients and controls. The underlying mechanism between viral insults and disease in autism remains unclear. Pathogenesis may result due to cellular damage induced as a result of immune responses aimed at eradicating an invading virus. Equally, impaired immune responses could increase susceptibility to infections in autistic children, or an inability to clear viruses leading to persistent infections that could then subvert immune function or act directly upon the CNS to elicit neurodevelopmental abnormalities. The study of pathogen-induced immune responses and the pattern of pre- and post-natal exposures on the developing neuronal system may provide interesting data.

### MUCOSAL IMMUNITY IN ASD PATIENTS

Gastrointestinal symptoms have been described in a number of ASD patients, in whom symptoms include abdominal pain, bloating, diarrhoea and constipation (Horvath *et al.*, 1999; Afzal *et al.*, 2003). The precise number of children with ASD who have gastrointestinal symptoms is not currently known, but some studies have estimated this to be between 18 and 40% (Baron-Cohen *et al.* 1996; 2000; Horvath *et al.*, 1999; Fombonne *et al.*, 2001). Reported gastrointestinal abnormalities include low activities of disaccharidase enzymes, defective sulphation of ingested phenolic amines (paracetamol), bacterial overgrowth with greater diversity and number of clostridial species, more numerous Paneth cells, increased intestinal permeability and a positive effects on behavioral cognition following dietary intervention (Knivsberg *et al.*, 1995; 2002; D'Eufemia *et al.*, 1996; Alberti *et al.*, 1999; Horvath *et al.*, 1999; Finegold *et al.*, 2002). Clinical and pathological studies have described an apparently characteristic gastrointestinal immunopathology in this subset of ASD children (Wakefield *et al.*, 1998, 2000; Furlano *et al.*, 2001; Torrente *et al.*, 2002), in which chronic ileo-colonic lymphoid nodular hyperplasia (LNH) and entero-colitis are key features. The mucosal lesion consists of a pan-enteric lymphocytic infiltrate, with a variable degree of acute inflammation and eosinophil infiltration (Wakefield *et al.*, 1998, 2000; Furlano *et al.*, 2001; Torrente *et al.*, 2002). Flow cytometric and immunohistochemical analyses of mucosal lymphocyte populations in ASD children have demonstrated qualitatively consistent abnormalities at different anatomical sites including stomach, duodenum, ileum and colon

(Furlano *et al.*, 2001; Torrente *et al.*, 2002; Ashwood *et al.*, 2003), indicating a relatively homogenous mucosal lymphocyte infiltrate. Although LNH is not an uncommon finding in children with allergies or immunodeficiency, there is increased frequency and severity in patients with autism (Wakefield *et al.*, 2002). In addition, findings of focal deposition of serum IgG from ASD children which co-localises with complement C1q on the basolateral enterocyte membrane, changes not seen in histologically normal and inflamed mucosa of developmentally normal children or children with cerebral palsy (Torrente *et al.*, 2002), and increased intestinal permeability in ASD (D'Eufemia *et al.*, 1996) is suggestive of an inflammatory process that may perturb the intestinal barrier function in ASD children. Co-localisation of immunoglobulin and complement components on the epithelial membrane have been found in both stomach and duodenal specimens and may be indicative of an autoimmune process directed against self-antigen contained within epithelial cells (Torrente *et al.*, 2002). Furthermore, increased basement membrane thickness and abnormal patterns of epithelial glycosaminoglycans have been seen in autistic children compared with controls and may be indicative of inflammatory degradation that could contribute to disruption of the intestinal barrier function (Furlano *et al.*, 2001).

While there is a great deal of speculation, the exact mechanism of how mucosal changes may influence autistic development or behavior is still not clear. It may be that antigens in the diet can cross the mucosa more easily through the disrupted intestinal barrier, where they cause local inflammatory reactions generating pro-inflammatory cytokine signals that interact with afferent neurons. Another possibility is that the failure to detoxify neuroactive antigens from the gut may lead to cognitive impairments. Similarly, increased passage of exorphins and/or opioids from the diet such as gliadomorphin and casomorphin into the body, where they can interact directly with the CNS, may play a role in inducing behavioral features of autism. In a further theory, reduced ileal absorption of vitamin $B_{12}$, a necessary cofactor for nerve myelogenesis and the formation of myelin (Wakefield *et al.*, 1998; Cornish, 2002), could lead to impaired nerve function, which again highlights how altered mucosal integrity may induce neuronal and behavioral changes. However, conclusive evidence to answer how disrupted intestinal mucosal functions affect neurodevelopment and the CNS is still necessary.

In peripheral blood, elevated pro-inflammatory cytokines have been reported to be produced upon stimulation with dietary proteins in autistic children compared with controls (Jyonouchi *et al.*, 2001). In some studies, circulating antibodies to food substances, namely casein and gliadin have been found (Reichelt *et al.*, 1990; Lucarelli *et al.*, 1995). However, these antibodies are also found with similar frequency in typically developing population. Furthermore, antibodies to neuronal specific antigens in the sera of children with autism could cross react with dietary peptides including milk butyrophilin, *streptococcus* M protein and *chlamydia pneumoniae* (Vojdani *et al.*, 2002), suggesting that bacterial infections and milk antigens may modulate the autoimmune process in autism. Interestingly, mucosal lymphocytes isolated from the duodenum, ileum and colon of ASD patients showed increased spontaneous production of pro-inflammatory intracellular cytokines, most notably TNFα, when compared with aged matched controls, including those with similar symptoms of constipation (Ashwood *et al.*, 2002, 2003). In addition, of particular interest was the inverse relationship between raised numbers of pro-inflammatory TNFα positive lymphocytes and decreased numbers of cells expressing the regulatory cytokine IL-10 in ASD children. These findings support the hypothesis that there is mucosal immune dysregulation with a pro-inflammatory lymphocyte cytokine profile, in ASD children. However, in a study that included one autistic child and two with ASD, macrophage release of monokines including IL-1β from *ex vivo* biopsy explant cultures into culture supernatant was not statistically different when compared with nine older children (Defelice *et al.*, 2003). These data may indicate differential roles for activated lymphocytes and antigen presenting cells in the entero-colitis associated with some autistic children. In an open-label trial, administration of vancomycin, an antibiotic that is poorly absorbed, resulted in objective cognitive improvements in autistic children (Sandler *et al.*, 2000), presumably due to treatment of intestinal dysbiosis. However, once administration of vancomycin was ceased, the patients cognitive functions regressed suggesting that the initial improvement was through beneficial effects on the intestinal pathology (Wakefield *et al.*, 2002). The exacerbation of gastrointestinal and behavioral symptoms in autism induced by certain foods, particularly those containing gluten and casein, has been shown through dietary intervention and their removal from the diet (Knivsberg *et al.*, 1995, 2002). Autistic children on gluten and casein free diets also showed significantly lower eosinophil infiltrate in intestinal biopsies compared with those on a conventional diet (Ashwood *et al.*, 2003). The significance of this finding is still unclear. However, it has been recently proposed that immune responses associated with allergy may contribute to the pathogenesis of autoimmune diseases of the CNS in both humans and in animal models (Pedotti *et al.*, 2003).

### IMMUNE THERAPY

Various studies have demonstrated that immunotherapy may be effective in the treatment of some children with autism (Gupta *et al.*, 1996; Plioplys, 1998; Gupta, 2000; Wank, 2002). As stated above, studies that have shown cognitive benefit from dietary intervention have been based upon the assumed removal of substrates for certain neuroactive opioid peptides present in the food

(Knivsberg *et al.*, 1995, 2002). Alternatively, although not exclusively, food antigens may provide a pro-inflammatory stimulus in some ASD children, as suggested by the data of Jyonouchi *et al.* (2001). Additionally, alterations in gut flora induced by dietary changes or by the administration of antibiotics may lead to beneficial effects. Further studies on immune based therapies including the administration of transfer factor, treatment with pentoxifylline, and intravenous immune globulin treatment have shown improvements in some autistic children (Gupta, 2000). However, these studies remain to be confirmed in larger placebo controlled, double-blinded, crossover studies with well matched control and subject groups. Furthermore, in depth behavioral assessments before and after treatment need to be performed.

### CONCLUSIONS

While there is a growing cognizance of immunological dysfunction in some ASD children, a great deal of research remains to be done. There are several aspects of the immune phenomenology thus far reported in ASD that require further investigation. Among these is the suggestion that patients with ASD have an altered or inappropriate response to viruses, the nature, extent and involvement of autoimmune processes in disease, and the importance of mucosal inflammation co-morbidity in some children. Each of these potentially pathological processes may be involved in the aetiology of autism in only a subset of children, or to varying degrees in all. Among the current and highly controversial data, there is much speculation, but little is truly known about the response of children with ASD to foreign antigens. The link between the immune and neurological systems is becoming increasingly well known. Products of the immune system play an important role in early neurodevelopment and can influence patterns of behavior. Bidirectional communication between the immune and nervous systems with many neuropeptides and neurotransmitters playing a role in both, is also of great interest. How the various immune abnormalities that have been reported in patients with ASD, are related to the development of neurologic changes is not yet known. In addition, exposure of the developing neuronal system to enhanced or aberrant immune responses during critical periods of development may not be uniform and may lead to the expression of different phenotypes in autistic patients, dependent upon rates of neuronal and immune development and genetic composition. So far, no clear signature patterns of cellular or cytokine responses have been found in autistic patients that can identify autism from other neurodevelopmental disorders, nor identify immunological findings to specific behavioral symptoms such as clinical behavioral regression. Large cohort studies on well defined patient and aged matched control groups will be needed to unravel the importance of the immune system in autism.

### *References*

Afzal, N., Murch, S., Thirrupathy, K., Berger, L., Fagbemi, A. and Heuschkel, R. (2003) "Constipation with acquired megarectum in children with autism", *Pediatrics* **112**(4), 939–942, Oct.

Ahlsen, G., Rosengren, L., Belfrage, M., *et al.* (1993) "Glial fibrillary acidic protein in the cerebrospinal fluid of children with autism and other neuropsychiatric disorders", *Biol. Psychiatry* **33**(10), 734–743.

Alberti, A., Pirrone, P., Elia, M., Waring, R.H. and Romano, C. (1999) "Sulphation Deficit in 'low functioning' autistic children: a pilot study", *Biol. Psychiatry* **46**, 420–424.

American Psychiatric Association (1994) Diagnostic and Statistical Manual of Mental Disorders, 4th Ed. (American Psychiatric Association, Washington, DC).

Anderson, G.M., Horne, W.C., Chatterjee, D. and Cohen, D.J. (1990) "The hyperserotonemia of autism", *Ann. N. Y. Acad. Sci.* **600**, 331–340.

Anderson, G.M., Gutknecht, L., Cohen, D.J., *et al.* (2002) "Serotonin transporter promoter variants in autism: functional effects and relationship to platelet hyperserotonemia", *Mol. Psychiatry* **7**, 831–836.

Ashwood, P., Walker-Smith, J.A., Murch, S.H. and Wakefield, A.J. (2002) "Pro-inflammatory cytokine production in the duodenal and colonic mucosa of children with autistic spectrum disorder (ASD) and a novel entero-colitis", *Gastroenterology* **122**, O617.

Ashwood, P., Anthony, A., Pellicier, A.A., Torrente, F., Walker-Smith, J.A. and Wakefield, A. (2003a) "Intestinal lymphoctye populations in children with regressive autism: evidence for extensive mucosal immunopathology", *J. Clin. Immunol.* **23**, 504–517.

Ashwood, P., Wakefield, A.J. and Murch, S.H. (2003b) "Increased spontaneous TNF and decreased IL-10 production in peripheral and ileal lymphocytes in autistic children", *JPGN* **36**(4), P22.

Bailey, A., Le Couteur, A., Gottesman, I., *et al.* (1995) "Autism as a strongly genetic disorder: evidence from a British twin study", *Psychol. Med.* **25**(1), 63–77.

Baird, G., Cass, H. and Slonims, V. (2003) "Diagnosis of autism", *BMJ* **327**(7413), 488–493.

Baron-Cohen, S., Cox, A., Baird, G., *et al.* (1996) "Psychological markers in the detection of autism in infancy in a large population", *Br. J. Psychiatry* **168**, 158–163.

Baron-Cohen, S., Wheelwright, S., Cox, A., *et al.* (2000) "Early identification of autism by the checklist for autism in toddlers", *J. Royal Soc. Med.* **93**, 521–525.

Bertrand, J., Mars, A., Boyle, C., Bove, F., Yeargin-Allsopp, M. and Decoufle, P. (2001) "Prevalence of autism in a United States population: the brick township, New Jersey, investigation", *Pediatrics* **108**, 1156–1161.

Binstock, T. (2001) "Intra-monocyte pathogens delineate autism subgroups", *Med. Hypothesis* **56**(4), 523–531.

Chakrabarti, S. and Fombonne, E. (2001) "Pervasive developmental disorders in preschool children", *JAMA* **285**(24), 3093–3099.

Chess, S., Fernandez, P. and Korn, S. (1978) "Behavioral consequences of congenital rubella", *J. Pediatr.* **93**, 699–703.

Chugani, D.C., Muzik, O., Behen, M., *et al.* (1999) "Developmental changes in brain serotonin synthesis capacity in autistic and nonautistic children", *Ann. Neurol.* **45**, 287–295.

Comi, A.M., Zimmerman, A.W., Frye, V.H., Law, P.A. and Peeden, J.N. (1999) "Familial clustering of autoimmune disorders and evaluation of medical risk factors in autism", *J. Child Neurol.* **14**, 388–394.

Connolly, A.M., Chez, M.G., Pestronk, A., Arnold, S.T., Mehta, S. and Deuel, R.K. (1999) "Serum autoantibodies to brain in Landau-Kleffner variant, autism, and other neurologic disorders", *J. Pediatr.* **134**(5), 607–613.

Conroy, J., Meally, E., Kearney, G., Fitzgerald, M., Gill, M. and Gallagher, L. (2004) "Serotonin transporter gene and autism: a haplotype analysis in an Irish autistic population", *Mol. Psychiatry*, 1–7.

Cook, E.H. and Leventhal, B.L. (1996) "The serotonin system in autism", *Curr. Opin. Pediatr.* **8**(4), 348–354.

Cook, E.H., Jr., Leventhal, B.L., Heller, W., Metz, J., Wainwright, M. and Freedman, D.X. (1990) "Autistic children and their first-degree relatives: relationships between serotonin and norepinephrine levels and intelligence", *J. Neuropsychiatry Clin. Neurosci.* **2**(3), 268–274.

Cook, E.H., Jr., Perry, B.D., Dawson, G., Wainwright, M.S. and Leventhal, B.L. (1993) "Receptor inhibition by immunoglobulins: specific inhibition by autistic children, their relatives, and control subjects", *J. Autism Dev. Disord.* **23**(1), 67–78.

Cornish, E. (2002) "Gluten and casein free diets in autism: a study of the effects on food choice and nutrition", *J. Hum. Nutr. Diet.* **15**, 261–269.

Croonenburghs, J., Deboutte, D. and Maes, M. (2002) "Pathophysiology of autism: current opinions", *Acta Neuropsychiatrica* **14**, 93–102.

D'Eufemia, P., Celli, M., Finocchiaro, R., *et al.* (1996) "Abnormal intestinal permeability in children with autism", *Acta Paediatr.* **85**(9), 1076–1079.

Dalton, P., Deacon, R., Blamire, A., *et al.* (2003) "Maternal antibodies associated with autism and language disorder", *Ann. Neurol.* **53**, 533–537.

Dantzer, R., Bluthe, R.M., Laye, S., *et al.* (1998) "Cytokines and sickness behavior", *Ann. N. Y. Acad. Sci.* **840**, 586–590.

Defelice, M.L., Ruchelli, E.D., Markowitz, J.E., *et al.* (2003) "Intestinal cytokines in children with pervasive developmental disorders", *Am. J. Gastroenterol.* **98**(8), 1777–1782.

Delneste, Y., Herbault, N. and Galea, B. (1999) "Vasoactive intestinal peptide synergizes with TNF-alpha in inducing human dendritic cell maturation", *J. Immunol.* **163**, 3071–3075.

Denney, D.R., Frei, B.W. and Gaffney, G.R. (1996) "Lymphocyte subsets and interleukin-2 receptors in autistic children", *J. Autism Dev. Disord.* **26**(1), 87–97.

Dunn, A.J., Wang, J. and Ando, T. (1999) "Effects of cytokines on cerebral neurotransmission. Comparison with the effects of stress", *Adv. Exp. Med. Biol.* **461**, 117–127.

Dunzendorfer, S., Kaser, A., Meierhofer, C., Tilg, H. and Wiedermann, C.J. (2000) "Dendritic cell migration in different micropore filter assays", *Immunol. Lett.* **71**, 5–11.

Engstrom, A.H., Ohlson, S., Stubbs, E.G., *et al.* (2003) "Decreeased expression of CD95 (FAS/APO-1) on CD4+T-lymphocytes from participants with autism", *J. Dev. Phys. Disabil.* **15**(2), 155–163.

Fatemi, S.H., Earle, J., Kanodia, R., *et al.* (2002) "Prenatal viral infection leads to pyramidal cell atrophy and macrocephaly in adulthood: implications for genesis of autism and schizophrenia", *Cell Mol. Neurobiol.* **22**(1), 25–33.

Ferguson, J.N., Young, L.J., Hearn, E.F., Matzuk, M.M., Insel, T.R. and Winslow, J.T. (2000) "Social amnesia in mice lacking the oxytocin. gene", *Nat. Genet.* **25**(3), 284–288.

Finegold, S.M., Molitoris, D., Song, Y., *et al.* (2002) "Gastrointestinal microflora studies in late-onset autism", *CID* **35**(Suppl. 1), S6–S16.

Fombonne, E. (1999) "The epidemiology of autism: A review", *Psychol. Med.* **29**, 769–786.

Fombonne, E., Simmons, H., Ford, T., Meltzer, H. and Goodman, R. (2001) "Prevalence of pervasive developmental disorders in the British nationwide survey of child mental health", *J. Am. Acad. Child Adolesc. Psychiatry* **40**, 820–827.

Furlano, R.I., Anthony, A., Day, R., *et al.* (2001) "Colonic CD8 and T-cell infiltration with epithelial damage in children with autism", *J. Pediatr.* **138**, 366–372.

Gimpi, G. and Fahrenholz, F. (2001) "The oxytocin receptor system: Structure. Function, and Regulation", *Physiol. Rev.* **81**(2), 629–683.

Gupta, S. (2000) "Immunological treatments for autism", *J. Autism Dev. Disord.* **30**(5), 475–479.

Gupta, S., Rimland, B. and Shilling, P.D. (1996) "Pentoxifylline: brief review and rationale for its possible use in the treatment of autism", *J. Child Neurol.* **11**(6), 501–504.

Gupta, S., Aggarwal, S., Rashanravan, B. and Lee, T. (1998) "Th1- and Th2-like cytokines in CD4+ and CD8+ T cells in autism", *J. Neuroimmunol.* **85**, 106–109.

Hans, A., Bajramovic, J.J., Syan, S., *et al.* (2004) "Persistent, non-cytolytic infection of neurons by Borna disease virus interferes with ERK 1/2 signalling and abrogates BDNF-induced synaptogenesis", *FASEB J.*, epub March.

Herman, B.H., Hammock, M.K., Arthur-Smith, A., *et al.* (1987) "Naltrexone decreases self-injurous behaviour", *Ann. Neurol.* **22**, 550–552.

Hornig, M., Weissenbock, H., Horscroft, N. and Lipkin, W.I. (1999) "An infection-based model of neurodevelopmental damage", *Proc. Natl Acad. Sci. USA* **96**, 12102–12107.

Horvath, K., Papadimitriou, J.C., Rabsztyn, A., Drachenberg, C. and Tildon, J.T. (1999) "Gastrointestinal abnormalities in children with autism", *J. Pediatr.* **135**, 559–563.

Jyonouchi, H., Sun, S. and Le, H. (2001) "Proinflammatory and regulatory cytokine production associated with innate and adaptive immune responses in children with autism spectrum disorders and developmental regression", *J. Neuroimmunol.* **120**(1–2), 170–179.

Kemper, T.L. and Bauman, M.L. (2002) "Neuropathology of infantile autism", *Mol. Psychiatry* **7**(Suppl. 2), S12–S13.

Klagge, I.M. and Scheider-Schaulies, S. (1999) "Virus interactions with dendritic cells", *J. Gen. Virol.* **80**, 832–833.

Knivsberg, A.-M., Reichelt, K.-L., Nodland, M. and Hoien, T. (1995) "Autistic syndromes and diet: a follow-up study", *Scand. J. Educ. Res.* **39**, 223–236.

Knivsberg, A.M., Reichelt, K.L., Hoien, T. and Nodland, M. (2002) "A Randomised, controlled study of dietary intervention in autistic syndromes", *Nutr. Neurosci.* **5**(4), 251–261.

Kozlovskaia, G.V., Kliushnik, T.P., Goriunova, A.V., Turkova, I.L., Kalinina, M.A. and Sergienko, N.S. (2000) "Nerve growth factor auto-antibodies in children with various forms of mental dysontogenesis and in schizophrenia high risk group", *Zh. Nevrol. Psikhiatr. Im. S. S. Korsakova* **100**, 50–52.

Larson, S.J. (2002) "Behavioral and motivational effects of immune-system activation", *J. Gen. Psychol.* **129**(4), 401–414.

Leboyer, M., Bouvard, M.P., Recasens, C., *et al.* (1994) "Difference between plasma N- and C-terminally directed beta-endorphin immunoreactivity in infantile autism", *Am. J. Psychiatry* **151**, 1797–1801.

Licinio, J., Kling, M.A. and Hauser, P. (1998) "Cytokines and brain function: relevance to interferon—a-induced mood and cognitive changes", *Semin. Oncol.* **25**(1), 30–38.

Lucarelli, S., Frediani, T., Zingoni, A.M., *et al.* (1995) "Food allergy and infantile autism", *Panminerva Med.* **37**, 137–141.

McDougle, C.J., Naylor, S.T., Cohen, D.J., Volkmar, F.R., Heninger, G.R. and Price, L.H. (1996a) "A double-blind, placebo-controlled study of fluvoxamine in adults with autistic disorder", *Arch. Gen. Psychiatry* **53**(11), 1001–1008.

McDougle, C.J., Naylor, S.T., Cohen, D.J., Aghajanian, G.K., Heninger, G.R. and Price, L.H. (1996b) "Effects of tryptophan depletion in drug-free adults with autistic disorder", *Arch. Gen. Psychiatry* **53**(11), 993–1000.

Menzebach, A., Hirsh, J., Hempelmann, G. and Welters, I.D. (2003) "Effects of endogenous and synthetic opioid peptides on neutrophil function *in vitro*", *Br. J. Anaesth.* **91**(4), 546–550.

Merali, Z., Lacosta, S. and Anisman, H. (1997) "Effects of interleukin-1 beta and mild stress on alterations of norepinephrine, dopamine and serotonin neurotransmission: A regional microdialysis study", *Brain Res.* **761**, 225–235.

Modahl, C., Green, L., Fein, D., *et al.* (1998) "Plasma oxytocin levels in autistic children", *Biol. Psychiatry* **43**(4), 270–277.

Mohankumar, P.S., Thyagarajan, S. and Quadri, S.K. (1991) "Interleukin-1 stimulates the release of dopamine and dihydroxy-phenylacetic acid from the hypothalamus *in vivo*", *Life Sci.* **48**, 925–930.

Murch, S.H., Anthony, A., Thomson, M., *et al.* (1999) "Ileo-colonic lymphoid nodular hyperplasia is associated with immunodeficiency in children with developmental disorders", *Gut* **44**, A127.

Nabi, B., Serajee, F.J., Chugani, D.C., Zhong, H. and Mahbubul Haq, A.H.M. (2004) "Association of Tryptophan 2,3 Dioxygenase gene polymorphism with autism", *Am. J. Med. Genetics* **125B**, 63–68.

Nelson, K.B., Grether, J.K., Croen, L.A., *et al.* (2001) "Neuropeptides and neurotrophins in neonatal blood of children with autism or mental retardation", *Ann. Neurol.* **49**(5), 597–606.

Nelson, P.T., Soma, L.A. and Lavi, E. (2002) "Microglia in diseases of the central nervous system", *Ann. Med.* **34**, 491–500.

Panksepp, J., Lensing, P., LeBoyer, M. and Bouvard, M.P. (1991) "Naltrexone and other potential pharmacological treatments of autism", *Brain Dysfunct.* **4**, 281–300.

Panoutsakopoulou, V. and Cantor, H. (2001) "On the relationship between viral infection and autoimmunity", *J. Autoimmun.* **16**, 341–345.

Pashenkov, M., Teleshova, N. and Link, H. (2003) "Inflammation in the central nervous system: the role for dendritic cells", *Brain Pathol.* **13**, 23–33.

Patterson, P.H. (2002) "Maternal infection: window on neuroimmune interactions in fetal brain development and mental illness", *Curr. Opin. Neurobiol.* **12**, 115–118.

Pedotti, R., De Vos, J.J., Steinman, L. and Galli, S.J. (2003) "Involvement of both 'allergic' and 'autoimmune' mechanisms in EAE, MS and other autoimmune diseases", *Trends Immunol.* **24**(9), 479–484.

Peterson, P.K., Molitor, T.W. and Chao, C.C. (1998) "The opioid-cytokine connection", *J. Neuroimmunol.* **83**(1–2), 63–69.

Plioplys, A.V. (1998) "Intravenous immunoglobulin treatment of children with autism", *J. Child Neurol.* **13**(2), 79–82.

Plioplys, A.V., Greaves, A., Kazemi, K. and Silverman, E. (1994) "Lymphocyte function in autism and Rett syndrome", *Neuropsychobiology* **29**(1), 12–16.

Ransohoff, R.M., Kivisakk, P. and Kidd, G. (2003) "Three or more routes for leukocyte migration into the central nervous system", *Nat. Rev.* **3**, 569–581.

Reichelt, K.L., Ekrem, J. and Scott, H. (1990) "Gluten, milk proteins and autism: dietary intervention effects on behavior and peptide secretion", *J. Appl. Nutr.* **42**, 1–11.

Risch, N., Spiker, D., Lotspeich, L., *et al.* (1999) "A genomic screen of autism: evidence for a multilocus etiology", *Am. J. Hum. Genet.* **65**(2), 493–507.

Rolf, L.H., Haarmann, F.Y., Grotemeyer, K.H. and Kehrer, H. (1993) "Serotonin and amino acid content in platelets of autistic children", *Acta Psychiatr. Scand.* **87**(5), 312–316.

Rosengren, L.E., Ahlsen, G., Belfrage, M., Gilberg, C., Haglid, K.G. and Hamberger, A. (1992) "A sensitive ELISA for glial fibrillary acidic protein: application in CSF of children", *J. Neurosci. Methods* **44**(2–3), 113–119.

Rumsey, J.M. and Ernst, M. (2000) "Functional neuroimaging of autistic disorders", *Ment. Retard. Dev. Disabil. Res. Rev.* **6**(3), 171–179.

Rutter, M. (2000) "Genetic studies of autism: from the 1970s into the millennium", *J. Abnorm. Child Psychol.* **28**(1), 3–14.

Sandler, R.H., Finegold, S.M. and Bolte, E.R. (2000) "Short-term benefit from oral vancomycin treatment of regressive-onset autism", *J. Child Neurol.* **15**(7), 429–435.

Sharp, B.M. (2003) "Opioid receptor expression and intracellular signaling by cells involved in host defense and immunity", *Adv. Exp. Med. Biol.* **521**, 98–105.

Shintani, F., Kanaba, S., Nakaki, T., *et al.* (1993) "Interleukin-1 beta augments release of norepinephrine, dopamine, and serotonin in the rat anterior hypoyhalamus", *J. Neurosci.* **13**, 3574–3581.

Singh, V.K. (1996) "Plasma increase of interleukin-12 and interferon-gamma. Pathological significance in autism", *J. Neuroimmunol.* **66**(1–2), 143–145.

Singh, V.K. and Rivas, W.H. (2004) "Prevalence of serum antibodies to caudate nucleus in autistic children", *Neurosci. Lett.* **355**(1–2), 53–56.

Singh, V.K., Warren, R.P., Odell, J.D., Warren, W.L. and Cole, P. (1993) "Antibodies to myelin basic protein in children with autistic behavior", *Brain Behav. Immun.* **7**, 97–103.

Singh, V.K., Singh, E.A. and Warren, R.P. (1997a) "Hyperserotoninemia and serotonin receptor antibodies in children with autism but not mental retardation", *Biol. Psychiatry* **41**(6), 753–755.

Singh, V.K., Warren, R., Averett, R. and Ghaziuddin, M. (1997b) "Circulating autoantibodies to neuronal and glial filament proteins in autism", *Pediatric. Neurol.* **17**, 88–90.

Singh, V.K., Lin, S.X. and Yang, V.C. (1998) "Serological association of measles virus and human herpesvirus-6 with brain autoantibodies in autism", *Clin. Immunol. Immunopathol.* **89**, 105–108.

Stubbs, G. (1995) "Interferonemia and autism", *J. Autism Dev. Disord.* **25**(1), 71–73.

Stubbs, E.G. and Crawford, M.L. (1977) "Depressed lymphocyte responsiveness in autistic children", *J. Autism Child Schizophr.* **7**, 49–55.

Sweeten, T.L., Bowyer, S.L., Posey, D.J., Halberstadt, G.M. and McDougle, C.J. (2003) "Increased prevalence of familial autoimmunity in probands with pervasive developmental disorders", *Pediatrics* **112**, 420.

Sweeten, T.L., Posey, D.J., Shankar, S. and McDougle, C.J. (2004) "High nitric oxide production in autistic disorder: a possible role for interferon-gamma", *Biol. Psychiatry* **55**(4), 434–437.

Szatmari, P. (1999) "Heterogeneity and the genetics of autism", *J. Psychiatry Neurosci.* **24**(2), 159–165.

Todd, R.D. and Ciaranello, R.D. (1985) "Demonstration of inter- and intraspecies differences in serotonin binding sites by antibodies from an autistic child", *Proc. Natl Acad. Sci. USA* **82**, 612–616.

Todd, R.D., Hickok, J.M., Anderson, G.M. and Cohen, D.J. (1988) "Antibrain antibodies in infantile autism", *Biol. Psychiatry* **23**(6), 644–647.

Torrente, F., Ashwood, P., Day, R., *et al.* (2002) "Small intestinal enteropathy with epithelial IgG and complement deposition in children with regressive autism", *Mol. Psychiatry* **7**, 375–382.

Uhlmann, V., Martin, C.M., Sheils, O., *et al.* (2002) "Potential viral pathogenic mechanism for new variant inflammatory bowel disease", *Mol. Pathol.* **55**, 84–90.

Vojdani, A., Campbell, A.W., Anyanwu, E., Kashanian, A., Bock, K., Vojdani, E. (2002) Antibodies to neuron-specific antigens in children with autism: possible cross reaction with encephalitogenic proteins from milk, *Chlamydia pneumoniae* and *Streptococcus* group A. 129: 168–177.

Volkmar, F.R. and Pauls, D. (2003) "Autism", *Lancet* **362**(9390), 1133–1141.

Von Boyen, G.B.T., Steinkamp, M., Remshagen, M., Schafer, K.H., Adler, G. and Kirsh, J. (2004) "Proinflammatory cytokines increase glial fibrillary acidic protein expression in enteric glia", *Gut* **53**, 222–228.

de Vos, A.F., van Meurs, M., Brok, H.P., *et al.* (2002) "Transfer of central nervous system autoantigens and presentation in secondary lymphoid organs", *J. Immunol.* **169**, 5415–5423.

Wakefield, A.J., Murch, S.H., Anthony, A., *et al.* (1998) "Ileal-lymphoid-nodular hyperplasia, non-specific colitis, and pervasive developmental disorder in children", *Lancet* **351**, 637–641.

Wakefield, A.J., Anthony, A., Murch, S.H., *et al.* (2000a) "Enterocolitis in children with developmental disorders", *Am. J. Gastroenterol.* **95**, 2285–2295.

Wakefield, A.J., Anthony, A., Murch, S.H., *et al.* (2000b) "Entero-colitis in children with developmental disorders", *Am. J. Gastroenterol.* **95**, 2285–2295.

Wakefield, A.J., Puleston, J., Montgomery, S.M., Anthony, A., O'Leary, J.J. and Murch, S.H. (2002) "Review article: the concept of entero-colonic encephalopathy, autism and opioid receptor ligands", *Aliment. Pharmacol. Ther.* **16**, 663–674.

Walker, J.S. (2003) "Anti-inflammatory effects of opioids", *Adv. Exp. Med. Biol.* **521**, 148–160.

Wank, R. (2002) "Schizophrenia and other mental disorders require long-term adoptive immunotherapy", *Med. Hypotheses* **59**(2), 154–158.

Warren, R.P., Margaretten, N.C., Pace, N.C. and Foster, A. (1986) "Immune abnormalities in patients with autism", *J. Autism Dev. Disord.* **16**, 189–197.

Warren, R.P., Foster, A. and Margaretten, N.C. (1987) "Reduced Natural Killer Cell Activity in Autism", *J. Am. Acad. Child Adol. Psychol.* **26**(3), 333–335.

Warren, R.P., Singh, V.K., Cole, P., *et al.* (1991) "Increased frequency of the null allele at the complement C4b locus in autism", *Clin. Exp. Immunol.* **83**, 438–440.

Warren, R.P., Burger, R.A., Odell, D., Torres, A.R. and Warren, W.L. (1994) "Decreased plasma concentrations of the C4B complement protein in autism", *Arch. Pediatr. Adolesc. Med.* **148**, 180–183.

Warren, R.P., Odell, J.D., Warren, W.L., *et al.* (1996) "Strong association of the third hypervariable region of HLA-DR beta 1 with autism", *J. Neuroimmunol.* **67**, 97–102.

Whitely, P., Rogers, J., Savery, D. and Shattock, P. (1999) "A gluten-free diet as an intervention for autism and associated spectrum disorders: preliminary findings", *Autism* **3**, 45–65.

Williams, J.R., Insel, T.R., Harbaugh, C.R. and Carter, C.S. (1994) "Oxytocin administered centrally facilitates formation of a partner preference in female prairie voles (Microtus ochrogaster)", *J. Neuroendocrinol.* **6**(3), 247–250.

Wilson, C.J., Finch, C.E. and Cohen, H.J. (2002) "Cytokines and cognition—The case for a head—to-toe inflammatory paradigm", *Geriatric Biosci.* **50**(12), 2041–2056.

Wing, L. and Potter, D. (2002) "The epidemiology of autistic spectrum disorders: is the prevalence rising?", *Ment. Retard. Dev. Disabil. Res. Rev.* **8**(3), 151–161.

Wirleitner, B., Neurauter, G., Schrocksnadel, K., Frick, B. and Fuchs, D. (2003) "Interferon-gamma-induced conversion of trptophan:immunologic and neuropsychiatric aspects", *Curr. Med. Chem.* **10**(16), 1581–1591.

Yamashita, Y., Fujimoto, C., Nakajima, E., Isagal, T. and Matsuishi, T. (2003) "Possible association between congenital cytomegalovirus infection and autistic disorder", *J. Aut. Dev. Dis.* **33**(4), 455–459.

Young, L.J., Lim, M.M., Gingrich, B. and Insel, T.R. (2001) "Cellular mechanisms of social attachment", *Horm. Behav.* **40**(2), 133–138.

Young, L.J., Pitkow, L.J. and Ferguson, J.N. (2002) "Neuropeptides and social behavior:animal models relevant to autism", *Mol. Psychiatry* **7**, S38–S39.

Zagon, I.S. (1987) "Endogenous opioids, opioid receptors and neuronal development", *NIDA Res. Monogr.* **78**, 61–71.

Zingarelli, G., Ellman, G., Hom, A., Wymore, M., Heldron, S. and Chicz-DeMet, A. (1992) "Clinical effects of naltrexone on autistic behavior", *Am. J. Ment. Retard.* **97**, 57–63.

